# The decreased platelet-to-lymphocyte ratio could predict a good prognosis in patients with oligometastatic colorectal cancer: a single-center cohort retrospective study

**DOI:** 10.1186/s12957-021-02406-z

**Published:** 2021-10-13

**Authors:** Xiaojuan Huang, Jin Cui, Xiaohui Li, Chao Liu, Jujie Sun, Jinbo Yue

**Affiliations:** 1 Clinical Medical College, Southwest Medical University, Luzhou, Sichuan China; 2 Department of Radiation Oncology, Shandong Cancer Hospital and Institute, Shandong First Medical University and Shandong Academy of Medical Sciences, Jinan, Shandong China; 3 Department of Radiation Oncology, Shandong Cancer Hospital, and Institute, Cheeloo College of Medicine, Shandong University, and Shandong Academy of Medical Sciences, Jinan, Shandong China; 4 Department of Pathology, Shandong Cancer Hospital and Institute, Shandong First Medical University and Shandong Academy of Medical Sciences, Jinan, Shandong China

**Keywords:** Colorectal cancer, Oligo-metastases, Inflammatory markers, Survival, PLR

## Abstract

**Background:**

Inflammation markers have an important effect on tumor proliferation, invasion, and metastasis. Oligometastatic disease (OMD) is an intermediate state between widespread metastases and locally confined disease, where curative strategies may be effective for some patients. We aimed to explore the predictive value of inflammatory markers in patients with oligometastatic colorectal cancer (OMCC) and build a nomogram to predict the prognosis of these patients.

**Methods:**

Two hundred nine patients with OMCC were retrospectively collected in this study. The Kaplan-Meier survival curves and Cox regression analysis were used to estimate overall survival (OS) and progression-free survival (PFS). A multivariate Cox analysis model was utilized to establish the nomogram. The concordance index (C-index), calibration curve, and receiver operating characteristics (ROC) were established to verify the validity and accuracy of the prediction model.

**Results:**

According to the multivariate analysis, decreased platelet-to-lymphocyte ratio (PLR) might independently improve OS in patients with OMCC (HR = 2.396, 95% CI 1.391–4.126, *P* = 0.002). Metastases of extra-regional lymph nodes indicated poor OS (HR = 2.472, 95% CI 1.247–4.903, *P* = 0.010). While the patients with early N stage had better OS (HR = 4.602, 95% CI 2.055–10.305, *P* = 0.001) and PFS (HR = 2.100, 95% CI 1.364–3.231, *P* = 0.007). Primary tumor resection (HR = 0.367, 95% CI 0.148–0.908, *P* = 0.030) and lower fibrinogen (HR = 2.254, 95% CI 1.246–4.078, *P* = 0.007) could significantly prolong the OS in patients with OMCC. PLR, metastases of extra-regional lymph nodes, N stage, primary tumor resection, and fibrinogen were used to make up the nomogram. The C-index and area under the curve (AUC) of the ROC in nomogram were 0.721 and 0.772 respectively for OS, showed good consistency between predictive probability of OS and actual survival.

**Conclusions:**

Decreased PLR could predict a good prognosis in patients with OMCC. The nomogram including inflammatory factors and clinicopathological markers was credible and accurate to predict survivals in patients with OMCC.

**Supplementary Information:**

The online version contains supplementary material available at 10.1186/s12957-021-02406-z.

## Introduction

Colorectal cancer (CRC) is the third most common cancer [1]. Metastasis occurs in 21% of patients with the first diagnosis of CRC and distant metastasis would occur in approximately 50% of these patients, resulting in a 5-year OS rate of less than 14% [2]. The liver is the most common site of metastasis, followed by lungs and other organs [3, 4].

In 1995, Hellman and Weichsel Baum proposed oligo-metastases, which are described as an intermediate state between extensive metastases and locally controlled disease [5, 6]. The diagnosis of oligometastatic colorectal cancer (OMCC) is dependent on imaging detection. According to available data, OMCC is defined as no more than 5 metastatic lesions in up to 3 sites [7]. The terms ‘synchronous’ and ‘metachronous’ are used to describe OMCC where metastases occur within or beyond 6 months after the diagnosis of the primary tumor [8]. The OMCC is indolent and moderate compared with the aggressiveness of widespread metastatic cancer, the prognosis of patients with OMCC could be significantly improved with aggressive treatments [9, 10]. And these patients could be potentially curable with surgical resection or other local therapies, which is rather promising than the fatal outcome of widespread metastases solid tumors [11]. But the survival of patients with OMCC varies greatly, a large proportion of patients who have received effective treatments fail to obtain satisfactory long-term survival because of recurrence, which lacks the identification of validated markers [12]. Studies showed that tumor-associated inflammation factors are related to the generation and development of tumors. Besides lymphocyte to monocyte ratio (LMR), neutrophil-to-lymphocyte ratio (NLR), and platelet-to lymphocyte ratio (PLR) of peripheral blood could represent the states of inflammatory and immune in patients [13, 14]. LMR, NLR, and PLR had been identified as prognostic predictors of malignancies development and survival in various tumors including lung cancer and breast cancer [15, 16]. Li and Xu [17, 18] built a nomogram of the composition of different risk factors, including inflammatory factors, which was reliable and practical for patients with colorectal cancer [19] and pancreatic cancer. But the evidence for the prognostic value of NLR, LMR, and PLR in patients with OMCC is limited. Therefore, it is crucial to explore the prognostic value of inflammatory factors on the survival in patients with OMCC to filter high-risk populations and select appropriate treatments for them.

In this study, we enrolled 209 patients with OMCC to investigate the prognostic effect of pretreatment LMR, NLR and PLR and established a nomogram with high validity and accuracy including inflammatory indicators and clinicopathological markers.

## Methods

### Patient selection

This was a single-center cohort retrospective study that analyzed 209 patients at Shandong Cancer Hospital and Institute, from January 2017 to June 2020. The selection criteria for patients were (1) the patient data on laboratory examination, imaging examination, and follow-up data was complete; (2) ultimate diagnosis was confirmed by histopathology; (3) the number of metastatic organs ≤3 and number of total metastatic lesions ≤5 [7]; (4) the metastases identified less 6months after the diagnosis of the primary tumor were defined as synchronous metastases [8]; (5) Eastern Cooperative Oncology Group (ECOG) < 2, patients were required to be > 18 years; (6) patients with other infections, hematologic disease, and other conditions that might affect markers of inflammation were excluded; and (7) patients with brain metastasis and peritoneal metastasis were excluded. The study was approved by the Institutional Research Ethics Committee of Shandong Cancer Hospital in Jinan, Shandong. The requirement for informed consent was waived because of the retrospective nature of the study. All methods were performed following the relevant guidelines and regulations.

### Data extraction and follow-up

The clinical data obtained from the electronic record system were as follows: age, gender, body mass index (BMI), primary tumor location, T stage, N stage, distribution of metastatic organs, number of involving sites, and treatments. The pretreatment laboratory data for the first diagnosis of oligo-metastases included NLR, PLR, LMR, white blood cell (WBC), fibrinogen carcinoembryonic antigen (CEA), and carbohydrate antigen 19–9 (CA19–9). The pathological stage of the primary tumor was depended on the Chinese Society of Clinical Oncology-Tumor Node Metastasis (CSCO-TNM) stage 2019 edition. All patients were followed up until September 2020 or the death of any case. Overall survival (OS), defined as the time length from the first diagnosis of OMCC to death of any case, was the primary endpoint of this study. The secondary endpoint was progression-free survival (PFS), which refers to the time from the first diagnosis of OMCC to metastatic lesions progression or death.

### Statistical analysis

The optimum cut-off value of NLR, PLR, and LMR was calculated by receiver operating curve (ROC) analysis according to OS. The chi-square test assessed the relevance between clinicopathologic factors and inflammatory ratios. The Kaplan-Meier curve estimated the OS and PFS. The Univariate analysis initially examined potential significant variables for OS and PFS, the multivariate analysis further estimated these significant variables and eventually determined independent prognostic factors for OS and PFS. Meanwhile, the Hazard ratios (HRs) and Confidence interval (CI) were also calculated.

The nomogram was developed based on the results of multivariate analysis [20, 21] and discrimination performance was examined by Harrell’s concordance index (C-index) and ROC analysis. Higher C-index represents better accuracy of the model. But a C-index of 0.5 is completely randomized, which indicates the predictive model is invalid [22]. The calibration curves were assessed by the bootstrap method with 1000 resamples, which could quantify the predictive validity of the nomogram. All statistical analyses were conducted on SPSS 26.0 and R 3.6.3 (http://www.r-project.org/). *P* < 0.05 was considered statistically significant.

## Results

### Baseline characteristics of patients

The median age of 209 patients was 60 (range 29–90) years. We observed liver-only metastases in 133 patients (63.3%), lung-only metastases in 79 patients (37.6%), liver-lung metastases in 12 patients (5.7%), and extra-regional lymph nodes metastases in 23 patients (11.0%). As for primary tumor, 28 (13.4%) patients had left-sided CRC, 179 (85.6%) had right-sided CRC. The N2 stage CRC was in 52 (24.9%) patients, the T4 stage CRC was in 103 (49.3%) patients (Table 1).

**Table 1:** Baseline characteristics of patients

Characteristics	Total patients, *n* (%)
Age (years) (median, range)	60 (29–90)
Gender	
Male	137 (65.6%)
Female	72 (34.4%)
BMI (kg/m^2^) (median, range)	
≤ 18.5	16 (7.6)
18.5–23.9	87 (41.4)
23.9–27	72 (34.3)
> 28	34 (16.7)
Timing of metastasis	
Synchronous	117 (55.7)
Metachronous	92 (44.3)
Liver-only metastases	133 (63.3)
Lung-only metastases	79 (37.6)
Liver-lung metastases	12 (5.7)
Extra-regional lymph nodes metastases	23 (11)
No. of involving sites	
1	90 (42.9)
≥ 2	119 (57.1)
Clinical T stage	
T2	17 (8.1)
T3	68 (32.5)
T4	103 (49.3)
Unknown	21 (10.1)
Clinical N stage	
N0	63 (30.1)
N1	69 (33.0)
N2	52 (24.9)
Unknown	25 (12.0)
Primary tumor location	
Left	179 (85.6)
Right	28 (13.4)
Unknown	2 (1)
WBC	
<4	25 (12.0)
4–10	171 (81.8)
> 10	13 (6.2)
LMR (median, range)	3.50 (0.44–69.30)
NLR (median, range)	2.36 (0.17–27.74)
PLR (median, range)	163.19 (0.00–475.86)
CA199(ng/ml)	
0–40	120 (57.4)
> 40	63 (30.2)
Unknown	26 (12.4)
CEA (ng/ml)	
0–5	74 (35.4)
> 5	120 (57.4)
Unknown	15 (7.2)
Fibrinogen(G/L)	
< 2	2 (1.0)
2–4	140 (67.0)
> 4	56 (26.7)
Unknown	11 (5.3)
Treatment	
Primary tumor resection	185 (88.1)
Lung resection	20 (9.5)
Liver resection	43 (20.5)
Interventional therapy	45 (21.5)
Radiotherapy	37 (17.7)
Chemotherapy	175 (83.7)
Targeted therapy	71 (34)

Abbreviations: *BMI* body mass index; *WBC* white blood cell; *LMR* lymphocyte-to-monocyte ratio; *NLR* neutrophil-to-lymphocyte ratio; *PLR* platelet to-lymphocyte ratio; *CA-199* carbohydrate antigen 19–9; *CEA* carcinoembryonic antigen

### Cut-off values of inflammatory markers

According to the maximum sum of specificity and sensitivity, we calculated the Youden index and obtained optimal cut-off values for NLR, LMR, and PLR were 3.57,3,97, and 208, respectively. CEA > 5 (ng/ml), CA19–9 > 40 (ng/ml), fibrinogen > 3.41 (G/L), and WBC > 10 (10^9/L) were classified into elevated-level groups based on the normal range.

### Relationships between LMR, NLR, and PLR and patients’ characteristics

We observed that 131(62.7%) patients were in the LMR-low group, 78 (37.3%) patients were in the LMR-high group, higher CEA level (*P* = 0.020) and CA-199 level (*P* = 0.019) were detected in the LMR-low group compared with the LMR-high group. 162 (77.5%) patients had decreased NLR which was significantly related with lower WBC (*P* < 0.001), fibrinogen (*P* = 0.016), and higher N-stage (*P* = 0.031). The males were more common in the elevated-NLR group than the low-NLR group (*P* = 0.005). Compared with the low-PLR group, patients in the high-PLR group significantly presented higher WBC (*P* = 0.038). Other clinical-related characteristics including age, BMI, the timing of metastasis, distribution of metastatic organs, number of involved sites, T stage, primary tumor location, treatments were comparable in different subgroups (all *P* > 0.05). More details were showed in Supplement Table 1.

### Relationships between LMR, NLR, PLR, and survival outcomes

**Fig. 1:**
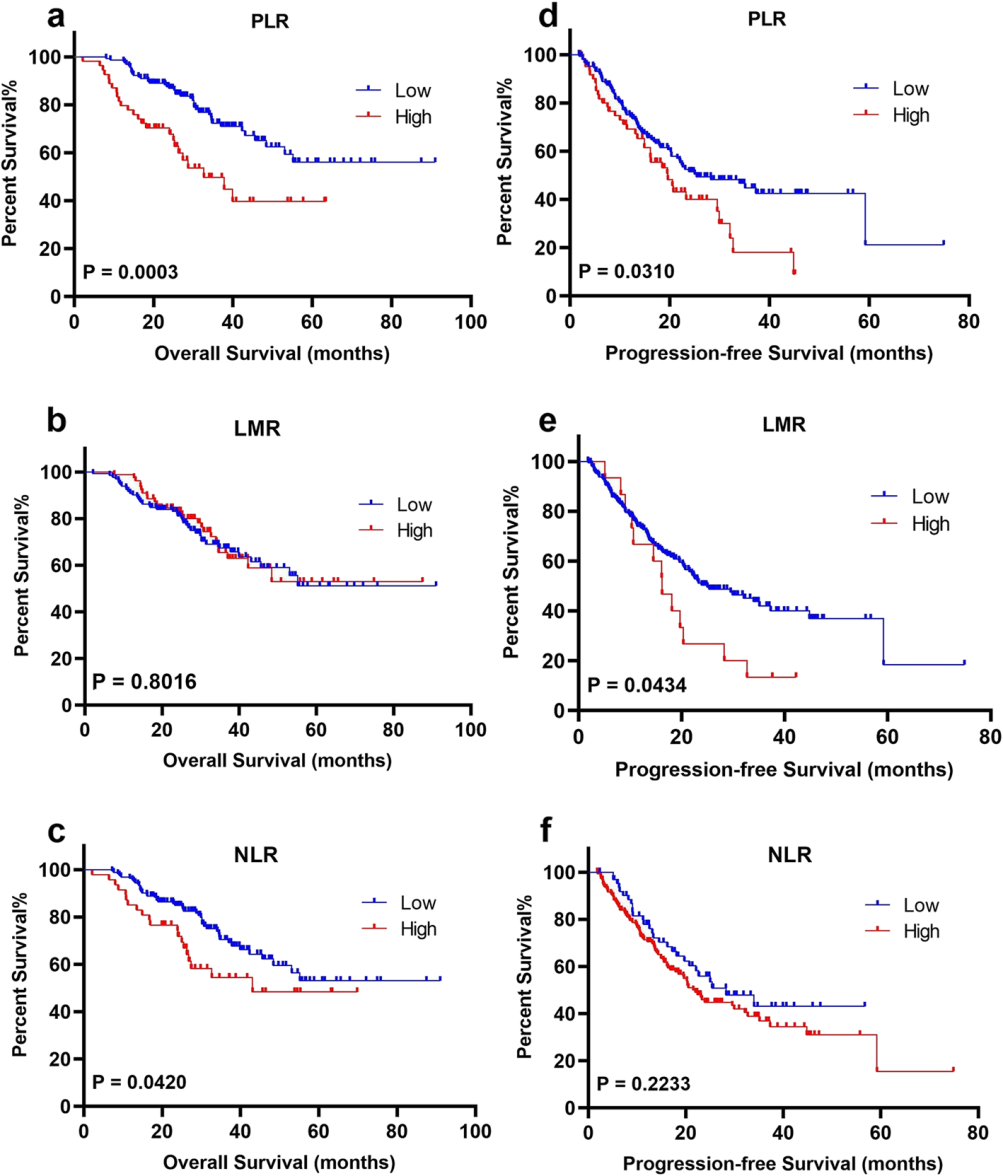
Kaplan-Meier curves of overall survival (OS) and progression-free survival (PFS) for platelet-to-lymphocyte ratio (PLR), lymphocyte-to-monocyte ratio (LMR), neutrophil-to-lymphocyte ratio (NLR). Notes: **a**, **b**, and **c** are the survival curves of PLR, LMR, and NLR for OS, respectively; **d**, **e**, and **f** are the survival curves of PLR, LMR, and NLR for PFS, respectively

The median follow-up was 32 (range 2–91) months. 142 (67.9%) patients were alive on the last follow-up, the cumulative 3-year and 5-year OS rates were 72.2% and 67.9%, respectively. One hundred forty-nine (71.2%) patients had evidence of cancer progression; the cumulative 3-year and 5-year PFS rates were 31.1% and 29.2%, respectively. Patients in the PLR-high group showed poorer OS (*P* = 0.0003) and PFS (*P* = 0.0310) than the PLR-low group, whereas low LMR only was associated with prolonged PFS (*P* = 0.0434). Compared with the NLR-low group, the OS for the NLR-high group was significantly improved (*P* = 0.0420). Figure 1 provided the relationships between LMR, NLR, PLR, and survival outcomes.

### Predictive accuracy of a nomogram based on pretreatment inflammatory markers and clinical pathology characteristics

The univariate analysis showed that extra-regional lymph nodes metastases (HR, 2.006, 95% CI 1.704–3.748, *P* = 0.029), more involving sites (HR, 1.797, 95% CI 1.803–3.748, *P* = 0.023), advanced N stage (HR, 4.195, 95% CI 1.972–8.924, *P* = 0.002), increased NLR (HR, 1.709, 95% CI 1.012–2.886, *P* = 0.045), and the high-level fibrinogen (HR, 2.777, 95% CI 1.593–4.840, *P* < 0.01) were associated with shorter OS. Primary tumor resection (HR, 0.501, 95% CI 0.261–0.960, *P* = 0.037), lung resection (HR, 0.242, 95% CI 0.059–0.987, *P* = 0.048), and reduced PLR (HR, 2.448, 95% CI 1.448–4.028, *P* < 0.001) were found statistically significant for longer OS. In addition, advanced N stage (HR, 2.083, 95% CI 1.353–3.206, *P* = 0.001) presented worse PFS (Supplement Table 2).

The multivariate analysis further revealed that extra-regional lymph nodes metastases (HR, 2.472, 95% CI 1.247–4.903, *P* = 0.010), early clinical N stage (HR, 4.602, 95% CI 2.055–10.305, *P* = 0.001), the low-level fibrinogen (HR, 2.254, 95% CI 1.246–4.078, *P* = 0.007), primary tumor resection (HR, 0.367, 95% CI 0.148–0.908, *P* = 0.03), and decreased PLR (HR, 2.396, 95% CI 1.391–4.126, *P* = 0.002) were related with good OS. Regarding PFS, advanced N stage (HR, 2.100, 95% CI 1.364–3.231, *P* = 0.007) independently predicted poor PFS (Table 2).

**Table 2:** Multivariate Cox analysis of factors associated with survival

Characteristics	OS					PFS				
	3-year (%)	Univariate		Multivariate		3-year (%)	Univariate		Multivariate	
		HR (95% CI)	*P*	HR (95% CI)	P		HR (95% CI)	*P*	HR (95% CI)	*P*
Extra-regional lymph nodes metastases										
No	68.7	Reference		Reference		26.7	Reference			
Yes	51.5	2.006 (1.704–3.748)	0.029	2.472 (1.247–4.903)	0.010	17.4	1.613 (0.994–2.617)	0.053		
Clinical N stage			0.002		0.001			0.001		0.007
N0	83.6	Reference		Reference		31.9	Reference		Reference	
N1	66.2	2.504 (1.164–5.387)	0.019	2.834 (1.256–6.396)	0.012	21.3	1.342 (0.888–2.028)	0.163	1.342 (0.888–2.028)	0.163
N2	48.3	4.195 (1.972–8.924)	<0.01	4.602 (2.055–10.305)	<0.01	17.4	2.083 (1.353–3.206)	0.001	2.100 (1.364–3.231)	0.001
Fibrinogen (G/L)										
≤3.41	80.2	Reference		Reference		27.6	Reference			
> 3.41	55.1	2.777 (1.593–4.840)	<0.01	2.254 (1.246–4.078)	0.007	24.9	1.008 (0.720–1.409)	0.965		
Primary tumor resection										
No	42.8	Reference		Reference		28.5	Reference			
Yes	69.1	0.501 (0.261–0.960)	0.037	0.367 (0.148–0.908)	0.030	24.9	1.385 (0.798–2.404)	0.246		
PLR										
≤208.48	72.3	Reference				28.8	Reference		Reference	
> 208.48	49.7	2.448 (1.448–4.028)	<0.01	2.396 (1.391–4.126)	0.002	13.4	1.414 (0.985–2.031)	0.061	1.371(0.953–1.972)	0.09

Abbreviations: *BMI* body mass index; *WBC* white blood cell; *LMR* lymphocyte-to-monocyte ratio; *NLR* neutrophil-to-lymphocyte ratio; *PLR* platelet to-lymphocyte ratio; *CA-199* carbohydrate antigen 19–9; *CEA* carcinoembryonic antigen; *HR* hazard ratio; *CI* confidence interval; *PFS* progression-free survival; *OS* overall survival

Based on the multivariate analysis, all statistically significant factors were identified to build the nomogram, including PLR, extra-regional lymph nodes metastases, clinical N stage, fibrinogen, and primary tumor resection (Fig.. 2). The C-index of the nomogram is 0.721 for 3-year OS (Fig. 3a) and 5-year OS (Fig. 3b), and the AUC of ROC in the nomogram was 0.772 (Fig. 4). The calibration and ROC curves showed no significant performance discrimination between predicted probability and real survival.

**Fig. 2:**
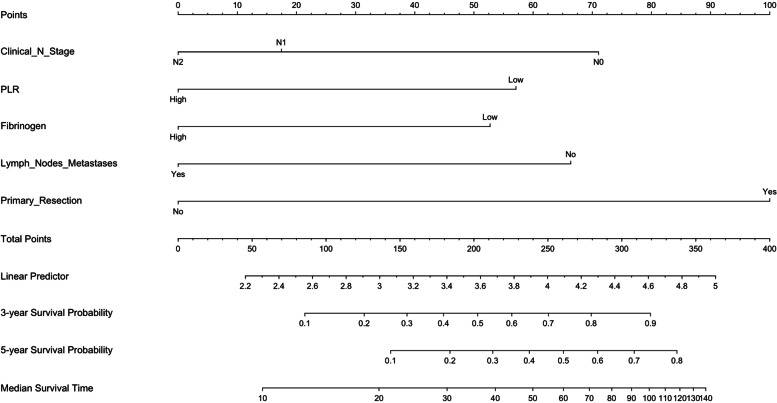
Nomogram for predicting the probability of 3-year, 5-year overall survival (OS), and median OS for patients with oligometastatic colorectal (OMCC). Notes To use the nomogram, the value of each patient was on each variable axis, and a line was drawn upward to determine the number of points received for each variable value. The sum of these numbers was on the total points axis. A line was drawn downward to the survival axes to determine the likelihood of 3-year, 5-year, and median survival time

**Fig. 3:**
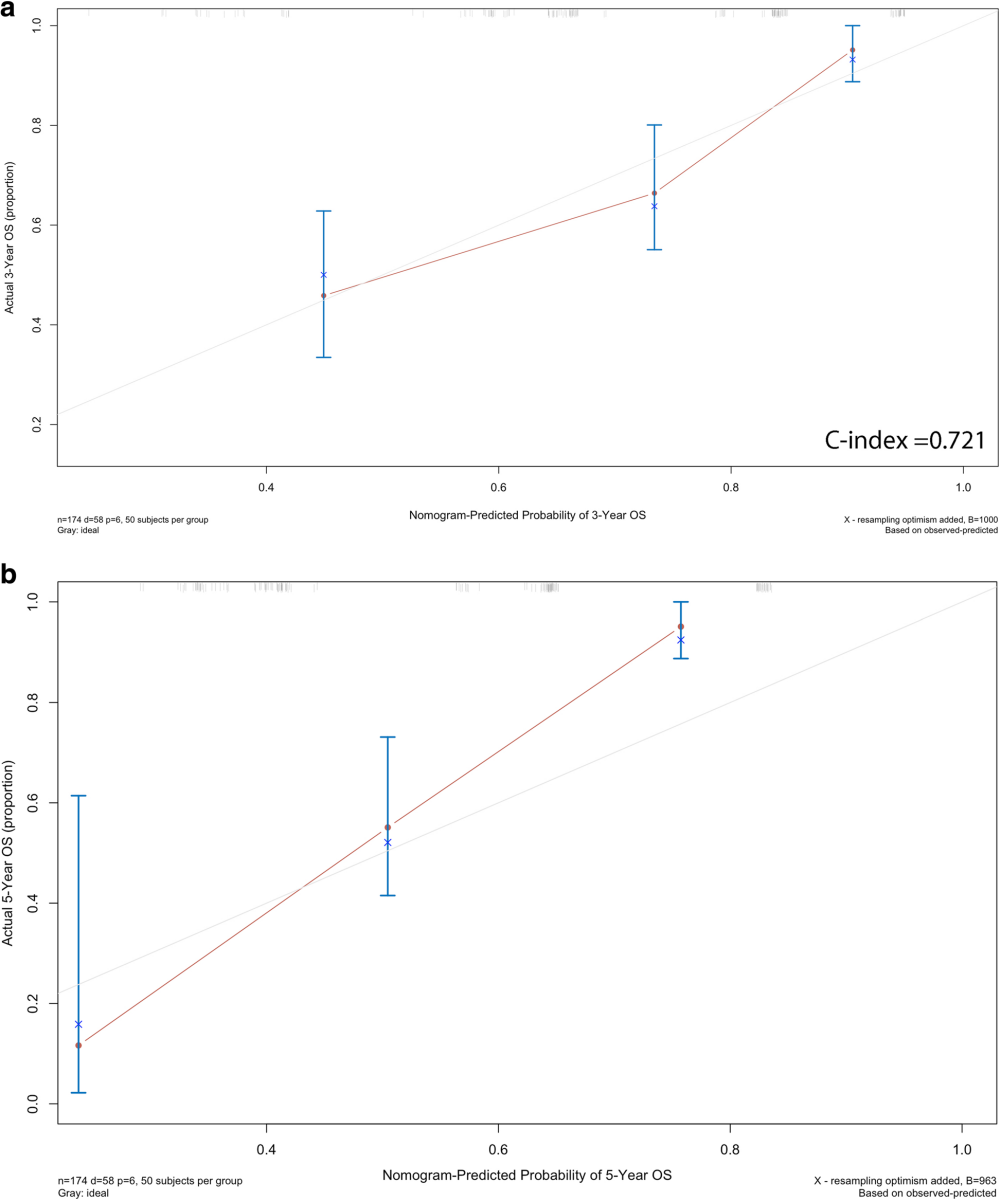
The calibration curve of nomograms for predicting patient survival at **a** 3 years and **b** 5 years. Notes: Nomogram-predicted probability of OS was plotted on the *x*-axis, actual OS was plotted on the *y*-axis

**Fig. 4:**
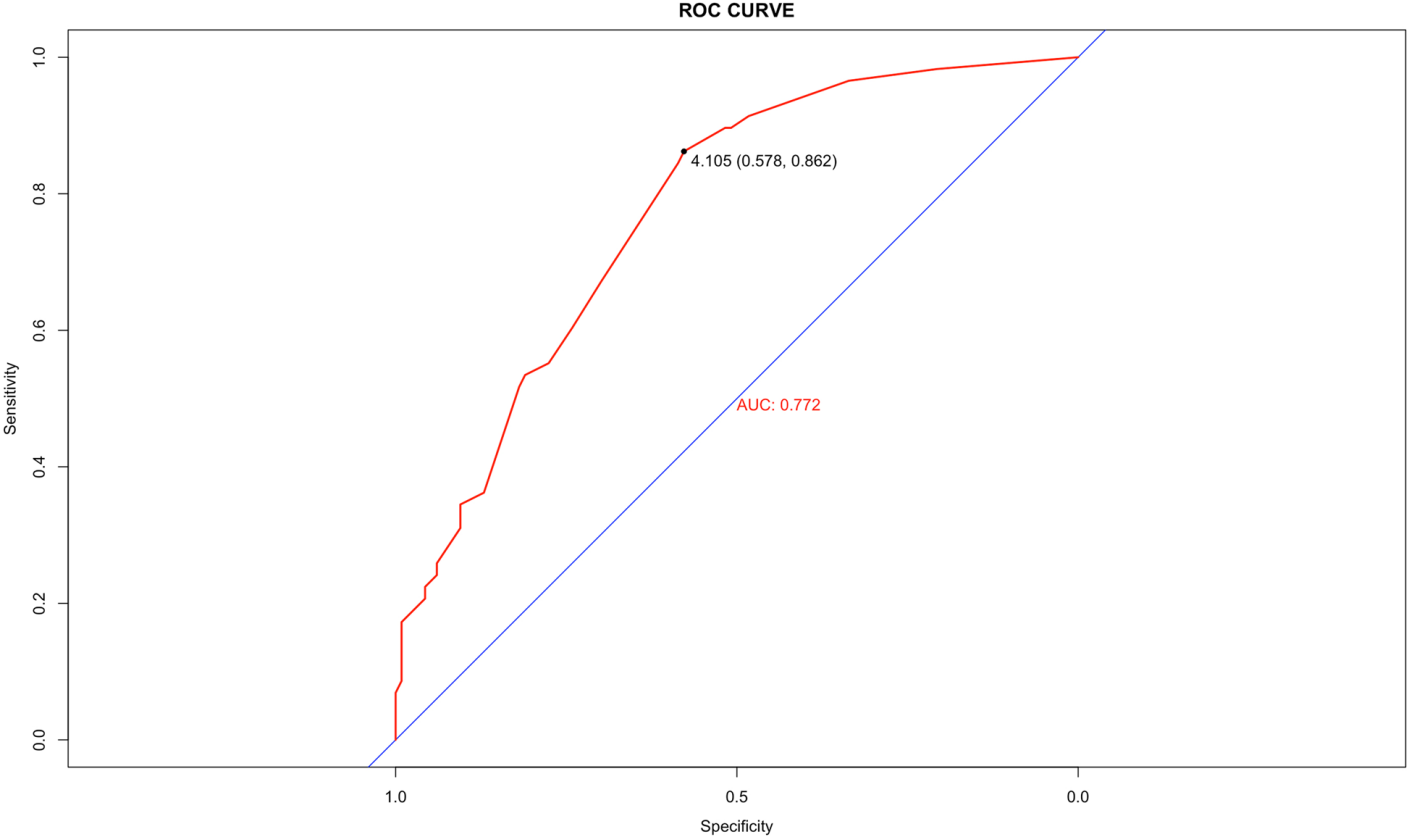
Predictive performance of nomogram for 3-year OS and 5-year OS by receiver operating characteristic (ROC) curves. Abbreviations: AUC, area under the curve

## Discussion

Dysregulated inflammatory response has been shown to have an essential impact on the activation and progression of tumors [23]. Inflammatory factors promote the proliferation, neovascularization, metastasis, invasion, and therapeutic resistance of tumor cells through various molecular mechanisms [24–26]. Peripheral inflammatory factors are of excellent research value due to their convenience, non-invasive features. NLR, LMR, and PLR have been considered as significant predictive markers in CRC including CRC pulmonary metastases and liver metastases [27–30]. However, the predictive value of inflammatory markers in patients with OMCC has not been evaluated. The current study demonstrated that the decreased PLR ratio could predict a good prognosis in patients with OMCC.

Previous studies demonstrated that cancerous tissue would be infiltrated by numerous neutrophils which significantly increased in peripheral blood of cancer patients [31, 32]. In the cancer microenvironment, TGF-β signaling can classify neutrophils into N1 and N2 phenotypes [33]. Due to metalloproteinases and vascular endothelial growth factor (VEGF) production, the N2 phenotype is related to tumor invasion, angiogenesis, and metastasis [34], while lymphocytes could promote antitumor immunity. Increased neutrophils or decreased lymphocytes led to the elevation of NLR, which was related to tumor development and patient death. Chen et al. [35] searched that elevated NLR would be recognized as the independent predictor for better OS and PFS. Interesting, in the present study, NLR was not an independent predictor. Considering the study of Chen et al. just collected patients with pulmonary-only synchronous metastases (PSOM), while the current study analyzed patients with liver-only metastases, lung-only metastases, and liver-lung metastases. Therefore, due to the bigger populations in our study involved, the conclusions may be more applicable to a broader range of patients.

A few studies suggested that platelets may promote the proliferation and transformation of tumor cells and be activated by tumor cells, which enlarged the recurrence and mortality risk of cancer [36]. Some researchers identified PLR as independent predictors of CRC patients [37]. In our study, our results further supported that increased PLR was a significant predictor of poor OS (HR, 2.396, 95% CI 1.391–4.126, *P* = 0.002). Different from OMCC, the strong association between PLR and survival in patients with non-invasive colorectal cancer was not found reported by Woo Jin Choi [38] and Huizhong Li [39]. Data concerning prognostic values of PLR in widespread metastatic colorectal were inadequate and controversial, but the latest study indicated that high PLR played a negative role on PFS [40]. These findings might help us to identify that PLR as a serum biomarker may be important to stratify patients with OMCC based on the current criteria. Moreover, results in the present study were in accord with conclusions from Silvestris and Ryuk [41, 42] indicating that high-level fibrinogen and advance N stage were independent factors of poor prognosis.

OMCC is an indolent and moderate decease state with vital clinical value in colorectal cancer and classical metastasis, which encouraged clinicians to adopt more radical treatment modalities instead of palliative care. Our study showed that primary tumor resection was independently related to better OS (HR, 0.367, 95% CI 0.148–0.908, *P* = 0.03). In contrast, the iPACS trial [43] showed that CRC patients with asymptomatic primary and concurrent unresectable metastases had no survival benefit from primary tumor resection. The reasons for the conflict in the two studies were the inclusion of patients with peritoneal metastases, which lead to poor prognosis. Moreover, the iPACS trial wasn’t finished, which resulted in the survival data in the trial was incomplete, with an indistinct line of the location of the primary tumor. So, the extrapolation of the conclusion of the iPACS trial was debatable. Lung resection and liver resection improved OS, but the results were only numerically increased without statistically increased (87.7% vs 64.4%, *P* = 0.333, 73.8% vs 65%, *P* = 0.131, respectively). Tomoichiro Hirosawa conducted a study that aimed to identify prognostic factors in CRC patients with isolated pulmonary metastases undergoing pulmonary resection, the results showed that pulmonary resection significantly improved 5-year survival compared with non-resection patients [44] (46.7% vs 3.9%, *P <* 0.0001). The reason for the inconsistency may be the incorporation of many patients (24.9%) with an advanced N stage, which was an indicator for the poor prognosis in CRC with lung resection [45]. Likewise, the OS of patients was ameliorated by interventional therapy and radiotherapy (77.4% vs 63.8%, *P* = 0.113, 69.1% vs 65.7%, *P* = 0.190) based on the univariate analysis. What was surprising was that system treatment diminished the survival of patients (64.7 vs 76.2%, *P* = 0.747). These results would indicate that patients with OMCC may benefit from offensive local treatments.

Vishal et al. have suggested that CEA, CA-199, and the primary tumor location are independent predictors for patients with CRC [46, 47]. Serum levels of CEA and CA-199 are recommended to be evaluated clinically, which plays a vital role in disease monitoring, efficacy evaluation [48]. The median of CEA and CA-199 in this study were 8.15 and 22.20, which were within the normal value range in patients with OMCC. Patients with a primary tumor on the right side had a poorer prognosis than on the left [49]. However, the locations of the primary tumor were not significantly associated with prognosis, which may be caused by most of the patients in the current study were rectal cancer which was classified as the left side. Contrary to expectations, this study demonstrated that targeted therapy could not significantly improve the survival of patients with OMCC. The possible reason was that some patients had KRAS or NRAS mutation types, which were not sensitive to targeted therapy.

The nomogram that made up of multiple clinical indicators and biological attributes aimed to predict a specific clinical outcome or the probability of a particular type of event based on the values of multiple variables. In this study, we created and validated the nomogram that including PLR, extra-regional lymph nodes metastases, clinical N stage, fibrinogen, and primary tumor resection. The C-index and AUC were 0.721 and 0.772 respectively, suggesting high accuracy and validity of the nomogram to predict prognosis in patients with OMCC.

There were a few limitations in the study. This study is a retrospective study with a small sample size from a single-center, which may cause type I error. And different treatments other than primary tumor resection were not statistically significant in the multivariate Cox analysis, which was a confounding factor that could not ignore. Overall, our findings need to be validated by multi-center prospective studies with a large sample size. Further large randomized controlled studies should be taken to identify the role of inflammatory markers.

## Conclusions

In conclusion, pretreatment PLR was an independent prognostic factor for OS in patients with OMCC. The elevated level of PLR was significantly associated with worse OS and PFS. We built the nomogram as a predictive model with high accuracy and validity to guide treatment and recurrence surveillance in patients with OMCC.

## Supplementary Information

**Additional file 1 **Supplement **Table S1** Relationships between LMR, NLR, and PLR and patients’ characteristics

**Additional file 2 **Supplement **Table S2** Univariate Cox analysis of factors associated with survival

## Data Availability

The data are not publicly available due to privacy and ethical restrictions.
