# Supplementary material for: The decreased platelet-to-lymphocyte ratio could predict a good prognosis in patients with oligometastatic colorectal cancer: a single-center cohort retrospective study
Source: World J Surg Oncol. 2021 Oct 13;19:297. doi: 10.1186/s12957-021-02406-z (PMC8513170; doi:10.1186/s12957-021-02406-z)
Supplement: Supplementary file 1 — Additional file 1 Supplement Table S1 Relationships between LMR, NLR, and PLR and patients’ characteristics [file 12957_2021_2406_MOESM1_ESM.docx]

Supplement table 1 Relationships between LMR, NLR, and PLR and patients’ characteristics.

| Characteristics | LMR | | | NLR | | | PLR | | |
| --- | --- | --- | --- | --- | --- | --- | --- | --- | --- |
|  | ≤3.97 | >3.97 | P | ≤3.57 | >3.57 | P | ≤208.48 | >208.48 | P |
|  | n=131, n (%) | n=78, n (%) |  | n=162, n (%) | n=47, n (%) |  | n=155, n (%) | n=54, n (%) |  |
| Age, (years) |  |  | 0.669 |  |  | 0.623 |  |  | 0.155 |
| ≤60 | 70 (53.4) | 39 (50.0) |  | 86 (53.1) | 23 (48.9) |  | 76 (49.0) | 33 (61.1) |  |
| >60 | 61 (46.6) | 39 (50.0) |  | 76 (46.9) | 24 (51.1) |  | 79 (51.0) | 21 (38.9) |  |
| Gender |  |  | 0.549 |  |  | 0.005 |  |  | 0.259 |
| Male | 88 (67.2) | 49 (62.8) |  | 98 (60.5) | 39 (83.0) |  | 105 (67.7) | 32 (59.3) |  |
| Female | 43 (32.8) | 29 (37.2) |  | 64 (39.5) | 8 (17.0) |  | 50 (32.3) | 22 (40.7) |  |
| BMI |  |  | 0.368 |  |  | 0.354 |  |  | 0.172 |
| ≤18.5 | 11 (8.4) | 5 (6.4) |  | 10 (6.2) | 6 (12.8) |  | 9 (5.8) | 7 (13.0) |  |
| 18.5-23.9 | 58 (44.2) | 29 (37.2) |  | 67 (41.4) | 20 (42.6) |  | 63 (40.6) | 24 (44.4) |  |
| 23.9-27 | 45 (34.4) | 27 (34.6) |  | 56 (34.6) | 16 (34.0) |  | 54 (34.8) | 18 (33.3) |  |
| >28 | 17 (13.0) | 17 (21.8) |  | 29 (17.8) | 5 (10.6) |  | 29 (18.7) | 5 (9.3) |  |
| Timing of metastasis |  |  | 1.000 |  |  | 0.505 |  |  | 0.428 |
| Synchronous | 73 (55.7) | 44 (56.4) |  | 93 (57.4) | 24 (51.1) |  | 84 (54.2) | 33 (61.1) |  |
| Metachronous | 58 (44.3) | 34 (34.6) |  | 69 (42.6) | 23 (48.9) |  | 71 (45.8) | 21 (38.9) |  |
| Liver-only metastases |  |  | 0.054 |  |  | 0.497 |  |  | 0.142 |
| No | 41 (31.3) | 35 (44.9) |  | 61 (37.7) | 15 (31.9) |  | 61 (39.4) | 15 (27.8) |  |
| Yes | 90 (68.7) | 43 (55.1) |  | 101 (62.3) | 32 (68.1) |  | 94 (60.6) | 39 (72.2) |  |
| Lung-only metastases |  |  | 0.188 |  |  | 0.865 |  |  | 0.745 |
| No | 86 (65.6) | 44 (56.4) |  | 100 (61.7) | 30 (63.8) |  | 95 (61.3) | 35 (64.8) |  |
| Yes | 45 (34.4) | 34 (43.6) |  | 62 (38.3) | 17 (36.2) |  | 60 (38.7) | 19 (35.2) |  |
| Liver-lung metastases |  |  | 0.765 |  |  | 1.000 |  |  | 0.512 |
| No | 124 (94.7) | 73 (93.6) |  | 153 (94.4) | 44 (93.6) |  | 147 (94.8) | 50 (92.6) |  |
| Yes | 7 (5.3) | 5 (6.4) |  | 9 (5.6) | 3 (6.4) |  | 8 (5.2) | 4 (7.4) |  |
| Extra-regional lymph nodes metastases |  |  | 0.824 |  |  | 0.610 |  |  | 0.802 |
| No | 116 (88.5) | 70 (89.7) |  | 143 (88.3) | 43 (91.5) |  | 137 (88.4) | 49 (90.7) |  |
| Yes | 15 (11.5) | 8 (10.3) |  | 19 (11.7) | 4 (8.5) |  | 18 (11.6) | 5 (9.3) |  |
| No. of involving sites |  |  | 0.248 |  |  | 0.182 |  |  | 0.111 |
| 1 | 52 (39.7) | 38 (48.7) |  | 74 (45.7) | 16 (34) |  | 72 (46.5) | 18 (33.3) |  |
| ≥2 | 79 (60.3) | 40 (51.3) |  | 88 (54.3) | 31 (66.0) |  | 83 (53.5) | 36 (66.7) |  |
| Clinical T stage |  |  | 0.476 |  |  | 0.234 |  |  |  |
| T2 | 13 (9.9) | 4 (5.2) |  | 13 (8.0) | 4 (8.5) |  | 15 (9.7) | 2 (3.7) |  |
| T3 | 45 (34.4) | 23 (29.5) |  | 57 (35.2) | 11 (23.4) |  | 49 (31.6) | 19 (35.2) |  |
| T4 | 61 (46.6) | 42 (53.8) |  | 74 (45.7) | 29 (61.7) |  | 74 (47.7) | 29 (53.7) |  |
| Unknown | 12 (9.1) | 9 (11.5) |  | 18 (11.1) | 3 (6.4) |  | 17 (11.0) | 4 (7.4) |  |
| Clinical N stage |  |  | 0.906 |  |  | 0.031 |  |  | 0.552 |
| N0 | 39 (29.8) | 24 (30.8) |  | 41 (25.3) | 22(46.8) |  | 50 (32.3) | 13 (24.1) |  |
| N1 | 44 (33.6) | 25 (32.1) |  | 58 (35.8) | 11 (23.4) |  | 52 (33.5) | 17 (31.5) |  |
| N2 | 31 (23.6) | 21 (26.9) |  | 44 (27.2) | 8(17.0) |  | 36 (23.2) | 16 (29.6) |  |
| Unknown | 17 (13.0) | 8 (10.2) |  | 19 (11.7) | 6 (12.8) |  | 17 (11.0) | 8 (14.8) |  |
| Primary tumor location |  |  | 1.000 |  |  | 0.299 |  |  | 0.163 |
| Left | 112 (85.5) | 67 (85.9) |  | 141 (87.0) | 38 (80.9) |  | 136 (87.7) | 43 (79.6) |  |
| Right | 18 (13.7) | 10 (12.8) |  | 20 (12.3) | 8 (17.0) |  | 17 (11.0) | 11 (20.4) |  |
| Unknown | 1 (0.8) | 1 (1.3) |  | 1 (0.6) | 1 (2.1) |  | 2 (1.3) | 0 (0.0) |  |
| WBC |  |  | 0.051 |  |  | ＜0.001 |  |  | 0.038 |
| ＜4 | 17 (13.0) | 8 (10.3) |  | 23 (14.2) | 2 (4.2) |  | 14 (9.0) | 11 (20.4) |  |
| 4-10 | 102 (77.9) | 69 (88.5) |  | 136 (84.0) | 35 (74.5) |  | 133 (85.8) | 38 (70.4) |  |
| >10 | 12 (9.2) | 1 (1.3) |  | 3 (1.9) | 10 (21.3) |  | 8 (5.2) | 5 (9.32) |  |
| CA199 (ng/ml) |  |  | 0.019 |  |  | 0.684 |  |  | 0.384 |
| 0-40 | 77 (58.8) | 43 (55.1) |  | 92 (56.8) | 28 (59.6) |  | 91 (58.7) | 29 (53.7) |  |
| >40 | 44 (33.6) | 19 (24.4) |  | 48 (29.6) | 15 (31.9) |  | 43 (27.7) | 20 (37.0) |  |
| Unknown | 10 (7.6) | 16 (20.5) |  | 22 (13.6) | 4(8.5) |  | 21 (13.6) | 5 (9.3) |  |
| CEA (ng/ml) |  |  | 0.020 |  |  | 0.395 |  |  | 0.949 |
| 0-5 | 41 (31.3) | 33 (42.3) |  | 61 (37.7) | 13 (27.6) |  | 54 (34.8) | 20 (37.0) |  |
| >5 | 84 (64.1) | 36 (46.2) |  | 89 (54.9) | 31 (66.0) |  | 90 (58.1) | 30 (55.6) |  |
| Unknown | 6 (4.6) | 9 (11.5) |  | 12 (7.4) | 3 (6.4) |  | 11 (7.1) | 4 (7.4) |  |
| Fibrinogen (G/L) |  |  | 0.149 |  |  | 0.016 |  |  | 0.345 |
| <2 | 1 (0.8) | 1(1.3) |  | 2 (1.2) | 0 (0.0) |  | 2 (1.3) | 0 (0.0) |  |
| 2-4 | 81 (61.8) | 59 (75.6) |  | 115(71.0) | 25 (53.2) |  | 107 (69) | 33 (61.1) |  |
| >4 | 41 (31.3) | 15 (19.3) |  | 35 (21.6) | 21 (44.7) |  | 37 (23.9) | 19 (35.2) |  |
| Unknown | 8 (6.1) | 3 (3.8) |  | 10 (6.2) | 1 (2.1) |  | 9 (5.8) | 2 (3.7) |  |
| Primary tumor resection |  |  | 1.000 |  |  | 0.607 |  |  | 0.457 |
| No | 15 (11.5) | 9 (11.5) |  | 20 (12.3) | 4 (8.5) |  | 16 (10.3) | 8 (14.8) |  |
| Yes | 116 (88.5) | 69 (88.5) |  | 142 (87.7) | 43 (91.5) |  | 139 (89.7) | 46 (85.2) |  |
| Lung resection |  |  | 0.474 |  |  | 0.086 |  |  | 0.605 |
| No | 120 (91.6) | 69 (88.5) |  | 143 (88.3) | 46 (97.9) |  | 139 (89.7) | 50 (92.6) |  |
| Yes | 11 (8.4) | 9 (11.5) |  | 19 (11.7) | 1 (2.1) |  | 16 (10.3) | 4 (7.4) |  |
| Liver resection |  |  | 1.000 |  |  | 0.682 |  |  | 0.328 |
| No | 104 (79.4) | 62 (79.5) |  | 130 (80.2) | 36 (76.6) |  | 126 (81.3) | 40 (74.1) |  |
| Yes | 27 (20.6) | 16 (20.5) |  | 32 (19.8) | 11 (23.4) |  | 29 (18.7) | 14 (25.9) |  |
| Interventional therapy |  |  | 0.731 |  |  | 1.000 |  |  | 0.341 |
| Yes | 27 (20.9) | 18 (23.1) |  | 35 (21.9) | 10 (21.3) |  | 36 (23.5) | 9 (16.7) |  |
| No | 102 (79.1) | 60 (76.9) |  | 125 (78.1) | 37(78.7) |  | 117 (76.5) | 45 (83.3) |  |
| Radiotherapy |  |  | 0.576 |  |  | 0.517 |  |  | 0.679 |
| Yes | 25 (19.2) | 12 (15.4) |  | 27 (16.8) | 10 (21.3) |  | 29 (18.8) | 8 (14.8) |  |
| No | 105 (80.8) | 66 (84.6) |  | 134 (83.2) | 37 (78.7) |  | 125 (81.2) | 46 (85.2) |  |
| Chemotherapy |  |  | 0.116 |  |  | 0.261 |  |  | 0.523 |
| Yes | 105 (80.8) | 70 (89.7) |  | 138 (85.7) | 37 (78.7) |  | 131 (85.1) | 44 (81.5) |  |
| No | 25 (19.2) | 8 (10.3) |  | 23 (14.3) | 10(21.3) |  | 23 (14.9) | 10 (18.5) |  |
| Targeted therapy |  |  | 0.881 |  |  | 0.382 |  |  | 0.740 |
| Yes | 44 (33.6) | 27 (34.6) |  | 58 (35.8) | 13 (27.7) |  | 54 (34.8) | 17 (31.5) |  |
| No | 87 (66.4) | 51 (65.4) |  | 104 (64.2) | 34 (72.3) |  | 101 (65.2) | 37 (68.5) |  |

**Abbreviations**: BMI, Body Mass Index; WBC, white blood cell; LMR, lymphocyte-to-monocyte ratio; NLR, neutrophil-to-lymphocyte ratio; PLR, platelet to-lymphocyte ratio; CA-199, carbohydrate antigen 19-9; CEA, carcinoembryonic antigen.
