# Supplementary material for: The decreased platelet-to-lymphocyte ratio could predict a good prognosis in patients with oligometastatic colorectal cancer: a single-center cohort retrospective study
Source: World J Surg Oncol. 2021 Oct 13;19:297. doi: 10.1186/s12957-021-02406-z (PMC8513170; doi:10.1186/s12957-021-02406-z)
Supplement: Supplementary file 2 — Additional file 2 Supplement Table S2 Univariate Cox analysis of factors associated with survival [file 12957_2021_2406_MOESM2_ESM.docx]

Supplement table 2 Univariate Cox analysis of factors associated with survival.

| Characteristics | OS | | | | | PFS | | | | |
| --- | --- | --- | --- | --- | --- | --- | --- | --- | --- | --- |
|  | 3-year (%) | Univariate | | Multivariate | | 3-year (%) | Univariate | | Multivariate | |
|  |  | HR (95%CI) | P | HR (95%CI) | P |  | HR (95%CI) | P | HR (95%CI) | P |
| Age, (years) |  |  |  |  |  |  |  |  |  |  |
| ≤60 | 72.4 | Reference |  |  |  | 28.8 | Reference |  |  |  |
| >60 | 60.1 | 1.434 (0.886-2.323) | 0.143 |  |  | 23.2 | 0.968 (0.701-1.338) | 0.846 |  |  |
| Gender |  |  |  |  |  |  |  | 0.826 |  |  |
| Male | 68.6 | Reference |  |  |  | 26.7 | Reference |  |  |  |
| Female | 63.0 | 0.832 (0.509-1.360) | 0.462 |  |  | 24.0 | 1.039 (0.740-1.459) |  |  |  |
| BMI |  |  | 0.545 |  |  |  |  | 0.667 |  |  |
| ≤18.5 | 56.3 | Reference |  |  |  | 25.0 | Reference |  |  |  |
| 18.5-23.9 | 62.0 | 1.954 (0.725-5.266) | 0.350 |  |  | 30.9 | 0.880 (0.472-1.642) | 0.688 |  |  |
| 23.9-27 | 71.1 | 1.315 (0.622-2.778) | 0.207 |  |  | 20.3 | 1.112 (0.595-2.076) | 0.739 |  |  |
| >28 | 74.8 | 1.126 (0.518-2.445) | 0.186 |  |  | 29.7 | 0.973 (0.480-1.972) | 0.940 |  |  |
| Timing of metastasis |  |  |  |  |  |  |  |  |  |  |
| Synchronous | 59.1 | Reference |  |  |  | 23.5 | Reference |  |  |  |
| Metachronous | 75.9 | 0.670 (0.408-1.101) | 0.114 |  |  | 28.2 | 1.041 (0.752-1.441) | 0.809 |  |  |
| Liver-only metastases |  |  |  |  |  |  |  |  |  |  |
| No | 69.1 | Reference |  |  |  | 23.2 | Reference |  |  |  |
| Yes | 66.8 | 0.981 (0.598-1.610) | 0.940 |  |  | 27.1 | 0.892 (0.640-1.244) | 0.501 |  |  |
| Lung-only metastases |  |  |  |  |  |  |  |  |  |  |
| No | 66.4 | Reference |  |  |  | 25.7 | Reference |  |  |  |
| Yes | 66.9 | 0.989 (0.599-1.633) | 0.966 |  |  | 25.9 | 1.036 (0.744-1.443) | 0.833 |  |  |
| Liver-lung metastases |  |  |  |  |  |  |  |  |  |  |
| No | 66.4 | Reference |  |  |  | 26.1 | Reference |  |  |  |
| Yes | 66.9 | 1.746 (0.699-4.362) | 0.233 |  |  | 16.7 | 1.300 (0.683-2.473) | 0.424 |  |  |
| Extra-regional lymph nodes metastases |  |  |  |  |  |  |  |  |  |  |
| No | 68.7 | Reference |  | Reference |  | 26.7 | Reference |  |  |  |
| Yes | 51.5 | 2.006 (1.704-3.748) | 0.029 | 2.472 (1.247-4.903) | 0.010 | 17.4 | 1.613 (0.994-2.617) | 0.053 |  |  |
| No. of involving sites |  |  |  |  |  |  |  |  |  |  |
| 1 | 79 | Reference |  | Reference |  | 30.4 | Reference |  |  |  |
| ≥2 | 55.7 | 1.797 (1.083-2.982) | 0.023 | 1.370 (0.806-2.326) | 0.244 | 21.4 | 1.304 (0.940-1.809) | 0.111 |  |  |
| Clinical T stage |  |  | 0.425 |  |  |  |  | 0.135 |  |  |
| T2 | 85.6 | Reference |  |  |  | 52.9 | Reference |  |  |  |
| T3 | 60.7 | 3.080 (0.734-12.994) | 0.126 |  |  | 17.9 | 1.849 (0.910-3.754) | 0.089 |  |  |
| T4 | 67.6 | 2.453 (0.587-10.242) | 0.219 |  |  | 24.8 | 1.655 (0.828-3.307) | 0.154 |  |  |
| Clinical N stage |  |  | 0.002 |  | 0.001 |  |  | 0.001 |  | 0.007 |
| N0 | 83.6 | Reference |  | Reference |  | 31.9 | Reference |  | Reference |  |
| N1 | 66.2 | 2.504 (1.164-5.387) | 0.019 | 2.834 (1.256-6.396) | 0.012 | 21.3 | 1.342 (0.888-2.028) | 0.163 | 1.342 (0.888-2.028) | 0.163 |
| N2 | 48.3 | 4.195 (1.972-8.924) | ＜0.001 | 4.602 (2.055-10.305) | ＜0.001 | 17.4 | 2.083 (1.353-3.206) | 0.001 | 2.100 (1.364-3.231) | 0.001 |
| Primary tumor location |  |  |  |  |  |  |  |  |  |  |
| Left | 65.9 | Reference |  |  |  | 25.8 | Reference |  |  |  |
| Right | 73.9 | 0.805 (0.368-1.763) | 0.588 |  |  | 23.8 | 1.373 (0.864-2.181) | 0.180 |  |  |
| WBC |  |  | 0.924 |  |  |  |  | 0.581 |  |  |
| ≤4 | 74.5 | Reference |  |  |  | 36.6 | Reference |  |  |  |
| 4-10 | 65.1 | 1.103 (0.332-3.669) | 0.825 |  |  | 23.1 | 1.281 (0.749-2.189) | 0.366 |  |  |
| >10 | 65.8 | 1.200 (0.433-3.319) | 0.873 |  |  | 32.3 | 1.033 (0.438-2.437) | 0.941 |  |  |
| CA199 (ng/ml) |  |  |  |  |  |  |  |  |  |  |
| 0-40 | 68.3 | Reference |  |  |  | 23.7 | Reference |  |  |  |
| >40 | 58.1 | 1.558 (0.929-2.612) | 0.093 |  |  | 29.1 | 0.941 (0.650-1.361) | 0.745 |  |  |
| CEA (ng/ml) |  |  |  |  |  |  |  |  |  |  |
| 0-5 | 74.1 | Reference |  |  |  | 24.4 | Reference |  |  |  |
| >5 | 61.6 | 1.259 (0.746-2.127) | 0.389 |  |  | 26.3 | 0.998 (0.707-1.407) | 0.989 |  |  |
| Fibrinogen (G/L) |  |  |  |  |  |  |  |  |  |  |
| ≤3.41 | 80.2 | Reference |  | Reference |  | 27.6 | Reference |  |  |  |
| >3.41 | 55.1 | 2.777 (1.593-4.840) | ＜0.001 | 2.254 (1.246-4.078) | 0.007 | 24.9 | 1.008 (0.720-1.409) | 0.965 |  |  |
| Primary tumor resection |  |  |  |  |  |  |  |  |  |  |
| No | 42.8 | Reference |  | Reference |  | 28.5 | Reference |  |  |  |
| Yes | 69.1 | 0.501 (0.261-0.960) | 0.037 | 0.367 (0.148-0.908) | 0.030 | 24.9 | 1.385 (0.798-2.404) | 0.246 |  |  |
| Lung resection |  |  |  |  |  |  |  |  |  |  |
| No | 64.4 | Reference |  | Reference |  | 25.7 | Reference |  |  |  |
| Yes | 87.7 | 0.242 (0.059-0.987) | 0.048 | 0.489 (0.115-2.081) | 0.333 | 24.8 | 0.970 (0.559-1.682) | 0.912 |  |  |
| Liver resection |  |  |  |  |  |  |  |  |  |  |
| No | 65 | Reference |  |  |  | 28.8 | Reference |  |  |  |
| Yes | 73.8 | 0.595 (0.304-1.166) | 0.131 |  |  | 8.1 | 1.251 (0.847-1.848) | 0.260 |  |  |
| Interventional therapy |  |  |  |  |  |  |  |  |  |  |
| No | 63.8 | Reference |  |  |  | 22 | Reference |  |  |  |
| Yes | 77.4 | 0.566 (0.280-1.144) | 0.113 |  |  | 41.2 | 0.709 (0.467-1.076) | 0.106 |  |  |
| Radiotherapy |  |  |  |  |  |  |  |  |  |  |
| No | 65.7 | Reference |  |  |  | 28.1 | Reference |  |  |  |
| Yes | 69.1 | 1.499 (0.832-2.524) | 0.190 |  |  | 15.0 | 1.358 (0.913-2.019) | 0.131 |  |  |
| Chemotherapy |  |  |  |  |  |  |  |  |  |  |
| No | 76.2 | Reference |  |  |  | 40 | Reference |  |  |  |
| Yes | 64.7 | 0.902 (0.483-1.686) | 0.747 |  |  | 22.6 | 1.504 (0.937-2.414) | 0.091 |  |  |
| Targeted therapy |  |  |  |  |  |  |  |  |  |  |
| No | 72.3 | Reference |  |  |  | 27.2 | Reference |  |  |  |
| Yes | 51.7 | 1.612 (0.979-2.655) | 0.060 |  |  | 22.5 | 1.198 (0.855-1.677) | 0.294 |  |  |
| LMR |  |  |  |  |  |  |  |  |  |  |
| ≤3.97 | 67.5 | Reference |  |  |  | 25.0 | Reference |  |  |  |
| >3.97 | 65.4 | 0.938 (0.571-1.539) | 0.800 |  |  | 26.9 | 0.847 (0.606-1.184) | 0.332 |  |  |
| NLR |  |  |  |  |  |  |  |  |  |  |
| ≤3.57 | 70.4 | Reference |  | Reference |  | 26.3 | Reference |  |  |  |
| >3.57 | 54.3 | 1.709 (1.012-2.886) | 0.045 | 1.420 (0.771-2.615) | 0.260 | 22.8 | 1.217 (0.836-1.772) | 0.306 |  |  |
| PLR |  |  |  |  |  |  |  |  |  |  |
| ≤208.48 | 72.3 | Reference |  |  |  | 28.8 | Reference |  | Reference |  |
| >208.48 | 49.7 | 2.448 (1.448-4.028) | ＜0.001 | 2.396 (1.391-4.126) | 0.002 | 13.4 | 1.414 (0.985-2.031) | 0.061 | 1.371(0.953-1.972) | 0.09 |

**Abbreviations**: BMI, Body Mass Index; WBC, white blood cell; LMR, lymphocyte-to-monocyte ratio; NLR, neutrophil-to-lymphocyte ratio; PLR, platelet to-lymphocyte ratio; CA-199, carbohydrate antigen 19-9; CEA, carcinoembryonic antigen; HR, hazard ratio; CI, confidence interval. PFS, progression-free survival; OS, overall survival.
